# The Impact of Psychological Flexibility on Psychological Well-Being in Adults With Obesity

**DOI:** 10.3389/fpsyg.2021.636933

**Published:** 2021-03-22

**Authors:** Anna Guerrini Usubini, Giorgia Varallo, Valentina Granese, Roberto Cattivelli, Simone Consoli, Ilaria Bastoni, Clarissa Volpi, Gianluca Castelnuovo, Enrico Molinari

**Affiliations:** ^1^Istituto Auxologico Italiano, Psychology Research Laboratory, Milan, Italy; ^2^Department of Psychology, Catholic University of Milan, Milan, Italy

**Keywords:** psychological flexibility, psychological well-being, health psychology, Acceptance and Commitment Therapy, obesity, rehabilitation

## Abstract

Obesity is a global health problem that affects both physical and psychological health and well-being. Psychological flexibility is one of the key components related to psychological health. This cross-sectional study aims to investigate the impact of psychological flexibility on psychological well-being in a sample of 220 individuals with obesity. Multivariate analysis was performed to investigate the role of psychological flexibility in explaining psychological well-being, controlling for confounding factors (sex, age, and Body Mass Index). According to the results, psychological flexibility significantly explained psychological well-being. Our study provides additional evidence of the impact of psychological flexibility on psychological well-being. It also provides further support for the importance of integrating psychological flexibility in the psychological interventions for obesity.

## Introduction

Obesity is a chronic health condition determined by excessive accumulation of adipose tissue, generally explained through the interaction of two conditions: excessive food intake and inadequate levels of physical activity (Castelnuovo et al., 2017). Obesity is a complex disease with a multifactorial etiology. Genetic, metabolic, as well as socio-cultural, behavioral, and environmental factors contribute to determining a condition of obesity (Curry, 2013; Boles et al., 2017). Also, both sexes and all ages are affected by obesity (World Health Organization Technical Report Series, 2000). Over the last fifty years, obesity has increased considerably, reaching pandemic levels. In 2014, more than 1 billion people worldwide were overweight. Of these, over 600 million meet the criteria for obesity based on Body Mass Index (BMI: Kg/m^2^ > 30) (World Health Organization Technical Report Series, 2000).

Obesity is considered a risk factor for many medical complications and comorbidities. Specifically, it impairs both physical and psychological well-being. It increases risks for metabolic, cardiovascular, and musculoskeletal diseases, such as type 2 diabetes, hypertension, and osteoarthritis as well as some types of cancer, dyslipidemia hypercholesterolemia and obstructive sleep apnea syndrome. Similarly, obesity and overweight are associated with a broad variety of psychological consequences such as depression, anxiety, eating disorders, lower quality of life, and lower self-esteem (Bray et al., 2017).

Most of the research on psychological flexibility has been conducted within the context of Acceptance and Commitment Therapy (ACT) (Hayes et al., 2006), a transdiagnostic approach raised in the third-wave Cognitive-behavioral Therapies (CBTs). According to ACT, acceptance of unpleasant experiences is more helpful than attempts to directly control inner events. Therefore, the aim of ACT is to promote psychological flexibility, defined as “*the ability to directly and openly contact experience in the present moment and persisting or changing behavior according to what the situation affords and one's personal goals and values*” (Hayes et al., 2006). Psychological flexibility is considered as a broad, overarching psychological process, resulting from the action of six key components: cognitive defusion (i.e., learning to distance from a person's thoughts); acceptance (i.e., an open attitude to painful inner experiences, including feelings, emotions, and thoughts); being in touch with the present moment; self as a context (i.e., contacting a stable sense of self regardless of one's personal experiences); finally, committed actions (i.e., pursuing actions or stable behaviors driven by personal values).

Numerous empirical studies have examined the efficacy of ACT in a variety of health conditions. Recently, a meta-analysis highlights that ACT was effective in reducing psychological distress and improving quality of life and a sense of hope in individuals with cancer (Zhao et al., 2021). In a randomized controlled trial, an ACT-based group intervention improved the quality of life, physical and psychological well-being in a sample of individuals with myocardial infarction (Ghahnaviyeh et al., 2020). Moreover, psychological flexibility was associated with health benefits in a range of clinical disorders (Powers et al., 2009; Kashdan and Rottenberg, 2010; Cattivelli et al., 2018) including anxiety and depressive symptomatology (Twohig and Levin, 2017) as well as chronic pain (Vowles and McCracken, 2008) and stress (Cristina et al., 2018). Together, these findings suggest that psychological flexibility is a key factor for the quality of life and psychological well-being (Biglan et al., 2008). The impact of ACT on psychological flexibility has been shown to help persons with obesity deal with self-stigma (Lillis et al., 2009), reduce binge eating (Lillis et al., 2011), and as a result of these changes improve quality of life and reduce weight (Lillis et al., 2009, 2011).

The study of psychological flexibility in individuals with obesity is still in its infancy. Few studies have examined this population. However, from previous evidence, it appears to be a promising aspect to be taken into account in the promotion of psychological well-being (Weineland et al., 2012; Cattivelli et al., 2018; Schumacher et al., 2019). It's therefore important to evaluate this aspect in individuals with obesity as well.

For this reason, the aim of the study is to evaluate the association between psychological flexibility and psychological well-being in a sample of individuals with obesity. In this study, we have focused on a definition of psychological well-being which highlights self-perceived health and psychological aspects of well-being, including both positive and negative intrapersonal affective or emotional states, emphasizes the individual, subjective appraisals (Diener, 1984; Dupuy, 1990; Grossi and Compare, 2014).

In the present cross-sectional study, we aimed at (1) evaluating the relationship between psychological flexibility and general psychological well-being in a sample of individuals affected by obesity; secondly, we aimed to (2) assess the role of psychological flexibility in explaining levels of psychological well-being. We hypothesized that (1) psychological flexibility would show a significant correlation with psychological general well-being; and that (2) psychological flexibility would explain significant and unique variance of indices of psychological general well-being.

## Materials and Methods

### Participants

The participants were 220 Italian obese adults 122 females (55,4%), 98 males (44,5%) aged between 18 and 87, who were consecutively recruited from the Istituto Auxologico Italiano, Ospedale S. Giuseppe, Piancavallo located in Northern Italy, at the beginning of a month-long hospitalization for weight loss. Only individuals with obesity (BMI ≥ 30) are eligible for hospitalization.

In-patients were eligible for the study if they met the following inclusion criteria: (1) age > 18 years; (2) BMI (Kg/m2) > 30; (3) written and informed consent to participate. Patients were excluded from the study if they did not meet the criteria for obesity (BMI ≥ 30) and had: (1) severe psychiatric disturbance diagnosed with the criteria of the Diagnostic and Statistical Manual of Mental Disorders (DSM-5); (2) concurrent severe medical condition that could compromise the participation in the study (e.g., blindness, deafness, neurological disorder, physical disability).

### Measures

Demographic data (i.e., sex, age, work status, etc.) were collected using a self-report form. The BMI was determined by the physician who, when the patient was admitted to the hospitalization, carried out the assessment visit.

To evaluate the independent variable, we used the *Acceptance and Action Questionnaire* (AAQ-II) (Bond et al., 2011). The AAQ-II is composed of 10 items (e. g “*I am afraid of my feelings,” “I worry about not being able to control my worries and feelings”)* rated from 1–7, on a 7-point Likert scale. It is the most widely used self-reported questionnaire that provides a measure of psychological flexibility. Higher total scores mean less flexibility, while lower total scores mean more flexibility. Scores range from a minimum of 0 to a maximum of 70. We used the Italian Validation (Pennato et al., 2013) that showed good psychometric properties, in agreement with the original version.

To evaluate the outcome, we used the Psychological General Well-Being Inventory (PGWBI) (Dupuy, 1990). The PGWBI is a validated measure widely used in clinical practice and research to provide a general subjective assessment of psychological well-being and health. We used the Italian validation of Grossi and colleagues (Grossi and Compare, 2014) that showed good psychometric properties in agreement with the original version. The questionnaire consists of 22 self-administered items rated on 6 point Likert scale ranged from 0 to 5 exploring six dimensions: anxiety (PGWBI_A; e.g., “*Have you been bothered by nervousness or your “nerves” during the past month?*”), depressed mood (PGWBI_D; e.g., “*I felt downhearted and blue during the past month*”), positive well-being (PGWBI_PWB; e.g., “*I felt cheerful, lighthearted during the past month*.”), self-control (PGWBI_SC; e.g., “*I was emotionally stable and sure of myself during the past month*”), general health (PGWBI_GH; e.g., “*How have you been feeling in general during the past month?*”), and vitality (PGWBI_V; e.g., “*I felt tired, worn out, used up, or exhausted during the past month*”). The six subscales consist of a minimum of three to a maximum of five items. The scores of all domains can be summarized to provide a summary score, which ranges from a minimum of 0 to a maximum of 110 points, representing the best achievable well-being.

### Procedures

At the beginning of the hospitalization, all patients underwent a clinical interview conducted by a clinical psychologist, to assess for current or pre-existing diagnosis of psychiatric disturbances. Patients diagnosed with psychiatric disorders were not selected for the study. According to eligibility criteria all recruited patients were asked to fill out the questionnaires abovementioned at the time of admission to the rehabilitation program. After providing informed and written consent to participate, questionnaires were administered in a research room, under the supervision of a psychologist involved in the research team. Data were collected from 1st December 2019 to 3rd March 2020.

The Medical Ethics Committee approved the study protocol and Informed Consent. All participants read, understood, and signed an informed consent document. All procedures on human subjects were conducted following the Helsinki Declaration of 1975, as revised in 1983.

### Statistical Analysis

The a-priori sample size was estimated for a Linear multiple regression: Fixed model, *R*^2^ deviation from zero using G.Power (version 3.1.9.4) (Faul et al., 2007) setting a small effect size (f2 = 0.10), an alpha of.05 and a power of.95, resulting in 191 participants.

Descriptive statistics have been conducted to describe the sample, detect missing values, and assess the normality of distributions. Descriptive statistics were calculated in terms of means and standard deviation for continuous variables, and in terms of frequencies and percentage for categorical variables.

Pearson's bivariate correlations have been computed to investigate the association between psychological flexibility and psychological well-being. A hierarchical regression analysis was then conducted to evaluate the contribution of the AAQ-II total score to the variance of the PGWBI total score. AAQ-II was set as the independent variable, controlling for sex, age and, BMI as possible confounding variables, and the total score of PGWBI was set as dependent variable. Confounding factors, that are likely to affect the main outcome were included in the first block; the AAQ-II total score was included in the second block. Δ*R*2 was used to evaluate the additional amount of variance in the dependent variable, which was accounted for by the AAQ-II score included in the second block compared to the first block including only confounding factors. Then, a MANOVA was performed to help protect against inflating the Type 1 error rate (Richard and Wichern, 2002). We set age, sex, BMI, and AAQ-II score as independent variables, and all subscales of the PGWBI as dependent variables.

Missing data lower than 5% was considered inconsequential (Schafer and Olsen, 1998). *P*-values <0.05 were considered statistically significant. Partial η-square values were interpreted according to Cohen (small = 0.01; medium = 0.06; large = 0.14; Cohen, 1977). The analyses were performed using Statistical Package for the Social Sciences (SPSS) version 26.

## Results

### Participants Characteristics

The sample was composed by 122 (55,5%) females and 98 males (44,5%) aged (in years) between 18 and 87 (*M* = 55.61; SD = 12.58), the average BMI (Kg/m^2^) was 43.66 ± 7.76. Almost half of the participants were married (48.9%) and had a high school degree (48.2%) and were employed (36.5%). Descriptive statistics of the sample were presented in Table 1.

**Table 1:** Descriptive statistics of the sample.

	**N (%)**	**Mean ± SD**	**Range**
Sex			
Male	98 (44.5)		
Female	122 (55.5)		
Age (in years)^*^	218	55.61 ± 12.58	18–87
Weight	220	119.27 ± 25.77	63.6–283
BMI (Kg/m^2^)	220	43.66 ± 7.76	
Educational level^*^			
Primary school	15 (6.9%)		
Secondary school	56 (25.7%)		
Higher school	105 (48.2%)		
Bachelor's degree	10 (4.6%)		
Master's degree	32 (14.6%)		
Marital Status^*^			
Single	66 (30.1%)		
Married	107 (48.9%)		
Divorced	26 (11.9%)		
Widowed	20 (9.1%)		
Work status^*^			
Student	5 (2.3%)		
Employed	80 (36.5%)		
Unemployed	31 (14.2%)		
Housewife	29 (13.2%)		
Retired	74 (33.8%)		
AAQ-II	220	33.98 (11.56)	10–61
PGWBI	220	66.08 (19.83)	12–106
Anxiety		15.81 (5.29)	1–25
Depression		11.50 (3.00)	0–15
Positive well-being		10.31 (4.16)	0–20
Self-control		10.38 (3.15)	2–15
General health		8.02 (2.87)	0–15
Vitality		10.19 (4.33)	0–20

***Note: AAQ-II: Acceptance and Action Questionnaire-II; PGWBI: Psychological General Well-being Index***.

* *Missing values lower than 5%*.

### Correlations

To assess the association between psychological flexibility and psychological well-being, correlational analyses were performed. AAQ-II resulted significantly and negatively related to all the PGWBI subscales. Correlations are presented in Table 2.

**Table 2:** Correlations.

	**PGWB_A**	**PGWB_D**	**PGWB_PWB**	**PGWB_SC**	**PGWB_GH**	**PGWB_V**	**PGWB_TOT**	**AAQ_II**
PGWB_A	—							
PGWB_D	0.786^*^	—						
PGWB_PWB	0.773^*^	0.786^*^	—					
PGWB_SC	0.689^*^	0.716^*^	0.748^*^	—				
PGWBI_GH	0.507^*^	0.543^*^	0.567^*^	0.510^*^	—			
PGWBI_V	0.663^*^	0.694^*^	0.780^*^	0.594^*^	0.679^*^	—		
PGWBI_TOT	0.884	0.874^*^	0.911^*^	0.818^*^	0.716^*^	0.864^*^	—	
AAQ-II	−0.422^*^	−0.495^*^	−0.524^*^	−0.571^*^	−0.248^*^	−0.358	−0.504^*^	—

*PGWBI_A, Psychological General Well-being Index_Anxiety; PGWBI_D: PGWBI, Psychological General Well-being Index_Depression; PGWBI_PWB: PGWBI, Psychological General Well-being Index_Positive well-being; PGWBI_SC: PGWBI, Psychological General Well-being Index_Self-Control; PGWBI_GH: PGWBI, Psychological General Well-being Index_General Helath; PGWBI_V: PGWBI, Psychological General Well-being Index_Vitality; PGWBI_TOT: PGWBI, Psychological General Well-being Index_total*.

* *p < 0.001*.

### Psychological General Well-Being

The full model of age, sex, BMI, AAQ-II score as the independent variables and PGWBI total score as the dependent variable was statistically significant, *R*^2^ = 0.265, Adjusted *R*^2^ = 0.270, [*F*(4, 202) = 20.06]; *p* < 0.001. The inclusion of the AAQ-II score, compared to the first block including only the confounding factors, explained about 24% additional variance, Δ*R*^2^ = 0.240; [*F*(1, 202) = 67.8]; *p* < 0.001. Only the AAQ-II score significantly explained the PGWBI total score (Table 3).

**Table 3:** Multivariable linear regression model examining the independent effect of demographic features and psychological flexibility (AAQ-II total score) on psychological well-being (PGWBI total score).

	**B**	**SE**	**β**	**95% CI**	***p*-value**
**Block 1: Confounding factors**					
Age	−0.063	0.096	−0.04	−0.254–0.128	0.516
Sex	4.407	2.398	0.222	−0.323–9.137	0.068
BMI	−0.142	0.177	−0.055	−0.445–0.161	0.357
**Block 2: Psychological flexibility**					
AAQ-II	−0.865	0.105	−0.506	−1.073–−0.658	<0.001^*^

*B, unstandardized beta; CI, confidence interval; AAQ-II, Acceptance and Action Questionnaire-II; BMI, Body Mass Index*.

* *p < 0.001*.

### Multivariate Analysis

We also conducted multivariate analysis to assess the role of psychological flexibility in explaining all the subscales of PGWBI, controlling for sex, age and BMI (Table 4). Gender served as fixed factor, while age, BMI and AAQ-II served as covariates. All the subscale of PGWBI were included as dependent variables. Only, the AAQ-II has a statistically significant effect on all PGWBI subscales, [*F*(6, 197) = 18.723]; *p* < 0.001; partial η^2^ = 0.363. Both sex [*F*(6, 197) = 1.634; *p* = 0.140; partial η^2^ = 0.047], and age [*F*(6, 197) = 2.051; *p* = 0.61; partial η^2^ = 0.59] and BMI [*F*(6, 197) = 1.382; *p* = 0.224; partial η^2^ = 0.040] resulted statistically non-significant.

**Table 4:** Parameter estimates.

**Variables**	**Parameter**	**B**	**Std. error**	**t**	**sig**	**95% confident intervals**		**Partial η^2^**
						**Lower**	**Upper**	
**PGWBI_A**	Intercept	22.161	2.885	7.681	0.000	16.472	27.850	0.226
	AAQ-II	−0.184	0.029	−6.272	0.000	−0.242	−0.126	0.163
	BMI	−0.009	0.043	−0.218	0.827	−0.094	0.075	0.000
	Age	0.020	0.027	0.736	0.463	−0.034	0.073	0.003
	Gender = 0	−1.477	0.671	−2.201	0.029	−2.800	−0.154	0.023
	Gender = 1	0						
**PGWBI_D**	Intercept	16.740	1.583	10.577	0.000	13.619	19.860	0.356
	AAQ-II	−0.127	0.016	−7.903	0.000	−0.159	−0.096	0.236
	BMI	−0.004	0.024	−0.152	0.880	−0.050	0.043	0.000
	Age	−0.007	0.015	−0.464	0.643	−0.036	0.022	0.001
	Gender = 0	−0.711	0.368	−1.933	0.055	−1.437	0.014	0.018
	Gender = 1	0						
**PGWBI_PWB**	Intercept	18.933	2.140	8.846	0.000	14.713	23.154	0.279
	AAQ-II	−0.193	0.022	−8.863	0.000	−0.236	−0.150	0.280
	BMI	−0.006	0.032	−0.189	0.850	−0.069	0.057	0.000
	Age	−0.024	0.020	−1.212	0.227	−0.064	0.015	0.007
	Gender = 0	−0.829	0.498	−1.665	0.097	−1.811	0.152	0.014
	Gender = 1	0						
**PGWBI_SC**	Intercept	16.504	1.544	10.687	0.000	13.459	19.549	0.361
	AAQ-II	−0.149	0.16	−9.491	0.000	−0.180	−0.118	0.308
	BMI	−0.012	0.023	−0.518	0.605	−0.057	0.033	0.001
	Age	0.000	0.015	0.013	0.990	−0.028	0.029	0.000
	Gender = 0	−1.018	0.359	−2.835	0.005	−1.726	−0.310	0.038
	Gender = 1	0						
**PGWBI_GH**	Intercept	14.968	1.666	8.982	0.000	11.683	18.254	
	AAQ-II	−0.067	0.017	−3.939	0.000	−0.100	−0.033	
	BMI	−0.059	0.025	−2.384	0.018	−0.108	−0.010	
	Age	−0.035	0.016	−2.225	0.027	−0.066	−0.004	
	Gender = 0	−0.273	0.388	−0.705	0.482	−1.037	0.491	
	Gender = 1	0						
**PGWBI_V**	Intercept	18.810	2.457	7.657	0.000	13.966	23.653	0.225
	AAQ-II	−0.141	0.025	−5.642	0.000	−0.191	−0.092	0.136
	BMI	−0.052	0.037	−1.431	0.154	−0.125	0.020	0.010
	Age	−0.024	0.023	−1.032	0.303	−0.069	0.022	0.005
	Gender = 0	−0.442	0.571	−0.773	0.440	−1.568	0.685	0.003
	Gender = 1	0						

*PGWBI_A, Psychological General Well-being_Anxiety; PGWBI_D, Psychological General Well-being_Depression; PGWBI_PWB, Psychological General Well-being_Positive well-being; PGWBI_SC, Psychological General Well-being_Self-Control; PGWBI_GH, Psychological General Well-being_General health; PGWBI_V, Psychological General Well-being_Vitality; AAQ-II, Acceptance and Action Questionnaire-II; BMI, Body Mass Index*.

As reported in the parameter estimates table below, AAQ-II significantly explained all subscales of the PGWBI. Sex was statistically significant in the Anxiety and Self-control subscales. The BMI and Age were statistically significant only in the General Health subscale. Parameter estimates have been reported in Table 4.

## Discussion

With respect to our first hypothesis, the results of the present study showed a statistically significant association between psychological flexibility and psychological well-being and health. The AAQ-II score resulted negatively and significantly correlated with psychological general well-being and all its separate dimensions (anxiety, depressive mood, positive well-being, general health, self-control, and vitality) in individuals with obesity. This means that less psychological flexibility is related to lower levels of psychological well-being.

With respect to our second hypothesis, according to the results psychological flexibility explained a statistically significant proportion of psychological well-being and its dimensions, when controlling for demographical and clinical factors, with proven explanatory value (Eichstaedt et al., 2020).

Our findings are consistent with the evidence supporting the psychological flexibility model developed by Hayes et al. (2006) in the field of ACT. According to this model, people who have higher levels of psychological well-being can establish an open and flexible contact with their own internal and external states (environmental) and engage themselves in committed actions related to personal values. Psychological flexibility has been strongly associated with several positive psychological outcomes, such as increased psychological well-being, reduced stress, anxiety, and depressive symptomatology (Bardeen et al., 2014; Francis et al., 2016; Tyndall et al., 2020). In opposition, decreased levels of psychological flexibility form the basis of psychological distress characterized by difficulties in emotional and behavioral regulation (Masuda and Tully, 2012).

Nowadays, current guidelines for obesity management strongly recommend providing comprehensive multi-professional and multidisciplinary interventions including exercise, diet, and behavioral or cognitive-behavioral therapy to promote healthy lifestyle change and psychological well-being. The current study has important clinical implications. Given the pandemic diffusion of obesity and in light of the well-documented poorer psychological health of individuals with obesity (Finger et al., 2020), it's important to address psychological factors related to obesity. To do so, identifying modifiable dimensions that influence psychological well-being, as psychological flexibility, should be the preliminary step to provide effective interventions.

Our study provides additional support to the existence of a link between psychological flexibility and well-being. This could be a valid promising result supporting the application of ACT-based intervention for individuals with obesity. Further studies are required to address the validity of such interventions, but some evidence has been already collected (Forman et al., 2013, 2016, 2019; Cattivelli et al., 2018).

Several limitations of the study must be addressed. First, all the data were cross-sectional, which warrants caution in drawing causal conclusions, although our goal was not to investigate causality. Secondly, the sample was composed exclusively of hospitalized individuals, which may induce a selection bias: thus, generalization to patients from different settings should be carefully done. Besides, our sample was composed of patients with a wide range of ages. Also, we did not investigate the presence of eating disorders in our sample. This may limit the generalizability of the results achieved.

On the other hand, this article has several strengths, which include the use of validated, reliable survey instruments, as well as an adequate sample size, and the fact that it presents data on a population on which there is still little research.

Longitudinal studies should be conducted to assess whether the improvement of psychological flexibility could have an effective impact on the psychological status of individuals with obesity and the rehabilitation outcomes, such as the adoption of long-standing healthy lifestyle habits. Future studies should also include individuals with obesity and comorbid eating disorders that were not considered in the present study.

## Conclusion

Achieving psychological well-being is one of the most relevant purposes psychological interventions aim for. Identifying the psychological processes that can help to protect psychological well-being is relevant. Findings from the current study suggest that psychological flexibility may be a helpful tool to identify those individuals most at risk of developing poor psychological well-being. Moreover, they provide additional evidence of the beneficial role of psychological flexibility on well-being and provides further support for the utility of enhancing psychological flexibility in the psychological interventions for obesity.

## Data Availability Statement

The raw data supporting the conclusions of this article will be made available by the authors, without undue reservation.

## Ethics Statement

The studies involving human participants were reviewed and approved by Istituto Auxologico Italiano. The patients/participants provided their written informed consent to participate in this study.

## Author Contributions

AG and GV conceived the study, planned the design, made a substantial contribution to the manuscript drafting, defined the statistical analysis, and establish the sample size for the study. VG, RC, SC, IB, and CV contributed greatly to the manuscript drafting. GC and EM read and approved the final manuscript. All authors contributed to the article and approved the submitted version.

## Conflict of Interest

The authors declare that the research was conducted in the absence of any commercial or financial relationships that could be construed as a potential conflict of interest.
